# Complete Genome Sequence of Hydrocarbon-Degrading Halotolerant Acinetobacter radioresistens DD78, Isolated from the Aconcagua River Mouth in Central Chile

**DOI:** 10.1128/MRA.00601-19

**Published:** 2019-08-15

**Authors:** Constanza C. Macaya, Valentina Méndez, Roberto E. Durán, Patricia Aguila-Torres, Francisco Salvà-Serra, Daniel Jaén-Luchoro, Edward R. B. Moore, Michael Seeger

**Affiliations:** a Molecular Microbiology and Environmental Biotechnology Laboratory, Department of Chemistry & Centro de Biotecnología Daniel Alkalay Lowitt, Universidad Técnica Federico Santa María, Valparaíso, Chile; b Laboratorio de Microbiología Molecular, Escuela de Tecnología Médica, Universidad Austral de Chile, Puerto Montt, Chile; c Department of Infectious Diseases, Institute for Biomedicine, Sahlgrenska Academy, University of Gothenburg, Gothenburg, Sweden; d Culture Collection University of Gothenburg (CCUG), Sahlgrenska University Hospital, Sahlgrenska Academy, University of Gothenburg, Gothenburg, Sweden; e Centre for Antibiotic Resistance Research (CARe), University of Gothenburg, Gothenburg, Sweden; f Microbiology, Department of Biology, University of the Balearic Islands, Palma de Mallorca, Spain; University of Southern California

## Abstract

Acinetobacter radioresistens strain DD78 (= CCUG 69565) is a soil hydrocarbon-degrading and biosurfactant-producing bacterium isolated from chronically crude oil-polluted soil of the Aconcagua River mouth in Chile. The 3.25-Mb *A. radioresistens* DD78 genome (41.8% GC content) was completely sequenced, with 4 replicons, 2,970 coding sequences, and 77 tRNAs.

## ANNOUNCEMENT

Acinetobacter species are aerobic Gram-negative coccobacilli present in soil, freshwater, sediment, polluted environments, and clinical samples (1, 2). A. baumannii, A. venetianus, and A. oleivorans degrade crude oil and alkanes (3, 4). A. radioresistens and A. calcoaceticus synthesize biosurfactants (e.g., alasan), improving hydrocarbon bioavailability (5, 6). Here, we report the complete genome sequence of *A. radioresistens* DD78 (CCUG_69565), a soil hydrocarbon-degrading and biosurfactant-producing bacterium from crude oil-polluted soil of the Aconcagua River mouth in Central Chile, which was isolated using enrichment in diesel (0.1% [vol/vol]) in Bushnell Hass mineral medium (7). Strain DD78 grew on *n-*hexadecane and diesel (7). DNA was extracted from Trypticase soy broth (TSB)-grown cells as described (8) and was purified using a Zymo Research DNA Clean & Concentrator-100 kit. Next-generation sequencing was performed using a PacBio Sequel v3 platform (Uppsala Genome Center, Sweden) with a SMRTbell library (average 20-kb insert library), obtaining 100,261 reads with an average length of 17,616 bp (raw read *N*_50_, 24,564 bp). The PacBio reads were trimmed and assembled into 5 contigs using SMRTLink v5 and the Hierarchical Genome Assembly Process v4.0 (HGAP4.0) with default parameters (9) and an expected 3.2-Mb genome size based on Acinetobacter genome sizes (10). The 5 contigs were reduced to 4 contigs with dotplot analysis using Gepard v1.40 with default settings (11). The DD78 genome (41.8% GC content, 246× coverage) consists of a 3,009,347-bp circular chromosome and three putative circular plasmids, pAr1 (88,584 bp), pAr2 (80,103 bp), and pAr3 (69,207 bp). Gene functional annotation using the NCBI Prokaryotic Genome Annotation Pipeline (PGAP) v4.6 (12) identified 2,970 coding sequences, 77 tRNAs, 7 rRNA operons, 4 noncoding RNAs (ncRNAs), and 1 CRISPR array. The Comprehensive Antibiotic Resistance Database (CARD) v3.0.0 (13) identified one antibiotic resistance gene (E3H47_05270), possessing 96.7% identity with oxacillinase OXA-239, from a clinical A. baumannii isolate (14). Genes encoding alasan protein components (*alnA*, *alnB*, and *alnC*: E3H47_11220, E3H47_09040, and E3H47_05485, respectively), proteins for osmoprotectant glycine betaine synthesis (*betIBA*: E3H47_10580, E3H47_10585, and E3H47_10590), and the osmoprotectant transporters osmo-dependent choline transporter, sodium/proline symporter, and aspartate/alanine antiporter (E3H47_06620, E3H47_06825, and E3H47_10585, respectively) were identified (>30% identity) with BLASTP using the Uniprot-KB/Swiss-Prot database. Arsenic and heavy metal (copper, cobalt, and cadmium) resistance genes are in pAr1. Catabolic genes encoding an alkane monooxygenase (*alkM*, E3H47_10210), a rubredoxin NAD(H) reductase/rubredoxin system (E3H47_10395 and E3H47_10400), and the catechol (*catBCA*: E3H47_07710, E3H47_07715, and E3H47_07720) and benzoate catabolic pathways (*benABCDE*: E3H47_07725, E3H47_07730, E3H47_07735, E3H47_07740, and E3H47_07745) are chromosomal. Comparative complete 16S rRNA gene sequence analysis showed a close relationship (99.7% identity) with *A. radioresistens* FO-1^T^ (CIP 103788^T^; GenBank accession number X81666) and <97.5% identity with other Acinetobacter species (15). Genome sequence analysis using average nucleotide identity based on BLAST (ANIb) (16) and using JSpeciesWS v3.0.2 (17) with *A. radioresistens* CIP 103788^T^, A. lwoffii ATCC 9957^T^, and *A. tandoii* CIP 107469^T^ showed ANIb values of 98.9%, 74.1%, and 73.6%, respectively, indicating that strain DD78 most likely belongs to the species *A. radioresistens*. The genome sequence of *A. radioresistens* DD78 provides essential data on alkane degradation, salt resistance, and biosurfactant production.

### Data availability.

The Acinetobacter radioresistens DD78 genome sequences have been deposited in DDBJ/ENA/GenBank under the accession number GCA_005519305 and the SRA accession number SRX5548971. The version discussed here is the first version. The BioProject number for the publicly available raw data is PRJNA528312.
